# The Prosocial Outgrowth of Filial Beliefs in Different Cultures: A Conditional Mediation Model Analysis

**DOI:** 10.3389/fpsyg.2021.748759

**Published:** 2021-10-21

**Authors:** Wang Zheng, Qingke Guo, Taian Huang, Jianli Lu, Chaoxiang Xie

**Affiliations:** ^1^Department of Psychology, Guangxi Normal University, Guilin, China; ^2^Department of Psychology, Shandong Normal University, Jinan, China

**Keywords:** filial piety, prosocial behavior, empathy, moral identity, gratitude, indebtedness, moderated mediation model

## Abstract

Filial piety is a concept originated from ancient China which contains norms of children’s feelings, attitudes, and behaviors toward their parents. The dual filial piety model (DFPM) differentiated two types of filial belief: reciprocal vs. authoritarian filial piety (RFP vs. AFP). Recent scholars suggest that the functions of filial piety may differ across cultures. This study examined the mediating effects of empathy, moral identity, gratitude, and sense of indebtedness in the relationship between filial piety and prosocial behavior (PB) and the moderating effects of nation. Questionnaires measuring filial piety, PB, moral identity, gratitude, and sense of indebtedness were administrated to Chinese and Indonesian participants. Moderated mediation modeling was conducted to analyze data. The results showed that empathy, moral identity, gratitude, and a sense of indebtedness have significant mediating effects in the association of filial piety and PB. And nation served as a moderator. (1) RFP could promote PB *via* enhanced empathy, moral identity, gratitude, and a sense of indebtedness, both among Chinese and Indonesian participants, while AFP did the same job only among Indonesian participants. (2) Among Chinese participants, AFP was not directly associated with PB, but was negatively associated with PB *via* reduced gratitude and a sense of indebtedness. (3) Nation (China vs. Indonesia) moderated the direct or indirect effect of RFP/AFP on PB, with RFP exerting stronger positive effects on outcome variables among Chinese (relative to Indonesian) participants and AFP exerting stronger positive effects on outcome variables among Indonesian (relative to Chinese) participants. These results showed that RFP can promote prosocial development by the cultivation of empathy, moral identity, gratitude, and a sense of indebtedness, regardless of whether the participants grew up in China or other cultural backgrounds. But the effect of AFP on PB was significantly conditioned by culture. This suggests that the function of RFP may be a cultural universal. However, the mechanisms that AFP influences PB can differ considerably across cultures. Findings of this study further indicate that filial piety beliefs may facilitate prosocial development in the ways conditioned by cultures.

## Introduction

Prosocial behavior (PB) refers to spontaneous and intentional behaviors that bring benefits to others, such as helping, comforting, cooperating, and caring for others (Eisenberg and Miller, 1987; Gross et al., 2017). PB can enhance the welfare of both the recipient and the actor. By acting prosocially, the helpers can build better interpersonal relationships with others, have greater life satisfaction and well-being, and get better academic performance (Caprara et al., 2000; Sun and Shek, 2010; Yang et al., 2017). Therefore, what factors contributing to PB and related influential mechanisms have become important topics in psychological research. Researchers believe that cultural value is an important factor that shapes individual prosocial development (Hofstede, 1980; Luria et al., 2015; Martí-Vilar et al., 2019). Socialization has been identified as a key mechanism by which cultural values can be transmitted from generation to generation, with family as the primary agent (McClintock et al., 1983). Consistent with this, numerous studies found that obligation to family plays an important role in promoting psychosocial development (Calderón-Tena et al., 2011; Knight et al., 2015). Familism, as a construct equivalent to filial piety that emphasizes responsibility and obedience to family, can facilitate prosocial outgrowth in different cultures (Schwartz et al., 2010).

Filial piety, as a primary ethical standard that affects Chinese people’s social behavior, is a cultural value that emphasizes respect, obedience, and respect for parents (Yeh, 2009). It has been a fundamental virtue and the core pillar of moral ideals in Chinese and other Confucianism-influenced societies. Filial piety has been the golden rule regulating parent-child relations for thousands of years (You et al., 2019; Brasher, 2021). Yeh and Bedford (2003) proposed the dual filial piety model (DFPM) to integrate the researches on filial piety in modern societies. DFPM can effectively address individual inferences in filial beliefs and can be used as the theoretical framework for cross-cultural comparisons (Shi and Wang, 2019; Tan et al., 2019; Beckert et al., 2020). It divides filial piety into two factors: reciprocal filial piety (RFP) and authoritarian filial piety (AFP). RFP focuses on close relationships formed by children and parents in long-term interactions, featured by children’s gratitude and love for their parents (Yeh and Bedford, 2004; Sun et al., 2019). On the contrary, AFP emphasizes family order and role norms that require the children to obey their parents (Yeh, 2006). Both types of filial piety (RFP and AFP) are advocated because they function interactively to enhance family cohesion. However, they have different effects on psychosocial functioning at the individual level (Yeh et al., 2013). This suggests that two types of filial piety may have different effects on PB.

According to the bioecological model (Bronfenbrenner and Morris, 2006), family is an environment where individuals interact frequently and directly, generating fundamental and tremendous impact on children’s psychological development. As a culture-specific value system, filial piety reflects one’s perception of social norms of obligation to parents (Chao and Tseng, 2002). Different types of filial piety correspond to different ways of parenting (Chen et al., 2015). Warm and rational parenting styles can cultivate children’s gratitude to their parents and consequently facilitates the development of children’s RFP (Huang and Yeh, 2013). Strict and demanding parenting styles lead to involuntary compliance with the parents’ wishes, which consequently leads to the development of children’s AFP (Huang and Yeh, 2013). Literature shows that warm, responsible, and supportive parenting can promote PB in children (Chen et al., 2015; Padilla-Walker et al., 2016; Kanacri et al., 2020), while strict and harsh parenting are negatively related to the child’s PB (Carlo et al., 2007; Padilla-Walker et al., 2016). Based on the above theorizing, we propose the following hypothesis:

*Hypothesis* 1: RFP positively predicts PB (H1a), while AFP negatively predicts PB (H1b).

### Mediating Roles of Empathy, Moral Identity, Gratitude, and Indebtedness

Many variables can account for the association of filial piety and PB. We selected empathy, moral identity, gratitude, and indebtedness as mechanisms because their mediating roles have not been statistically determined. These four variables are important moral dispositions that are responsible for individual differences in social behavior (Greenberg, 1980; Eisenberg and Miller, 1987; Hardy and Carlo, 2011; Bono et al., 2019). This study is interested in whether filial beliefs can foster the development of these dispositions thereby exerting influences on PB.

#### Empathy

As an important premise of filial piety attitude, family cohesion can promote the development of offspring’s empathy toward their parents (Cheung et al., 1994). And this kind of emotional care within the family is of great significance to the development of individual empathy (Main and Kho, 2020). In addition, researchers also found that the two types of filial piety beliefs (RFP and AFP) are significantly related to at least one of the two components of empathy (perspective-taking and empathic concern; Yeh and Bedford, 2003).

A large number of studies have shown that empathy can promote PB (Eisenberg and Miller, 1987; Telle and Pfister, 2015; Van der Graaff et al., 2018). Batson (1987) developed the empathy-altruism hypothesis, positing that when people perceive someone was suffering, they produce emotions, such as compassion and sympathy, which motivate prosocial actions to help the sufferer get rid of trouble. To date, the mediating role of empathy in the relationship between filial piety and PB has not been confirmed empirically. Therefore, we put forward the following hypothesis:

*Hypothesis*2: Empathy mediates the relationship between filial piety (RFP and AFP) and PB.

#### Moral Identity

Moral identity generally refers to the extent to which being an ethical person is important to one’s identity (Hardy and Carlo, 2011). According to Hart et al. (1999), family environment and parents are important factors that affect the formation of children’s moral identity. Several family factors, including family support, parenting style, parental involvement, and parental harshness, are important predictors of moral identity (Hart et al., 1998; Pratt et al., 2003; Hardy et al., 2010; Fatima et al., 2020). Therefore, we speculate that filial piety (both RFP and AFP) can predict moral identity, though no direct evidence on their relation is available.

As a construct bridging moral cognition and moral conduct, moral identity has been considered a significant predictor of PB (Aquino and Reed, 2002; Hardy, 2006; Hardy et al., 2015). Evidence shows that moral identity is associated with voluntary service, informal helping, empathy, and prosocial tendency (Aquino and Reed, 2002; Detert et al., 2008; Fatima et al., 2020). In view of these findings, we infer that filial piety is associated with PB *via* moral identity.

*Hypomthesis*3: Moral identity mediates the relationship between filial piety (RFP and AFP) and PB.

#### Gratitude

Family may be the first place where gratitude is cultivated (Scabini et al., 2006). In the family, individuals observe the sacrifice their parents have made for them and experience gratitude (Scabini, 2011). And they can extend these experiences to other interpersonal settings. Studies have found that children whose parents are warm, caring, and supportive (these parenting styles are closely associated with RFP) can develop a higher level of gratitude and apply it to other people (Lin, 2021). In contrast, children who are over controlled and interfered by their parents (these parenting styles are closely associated with AFP) tend to have lower levels of gratitude (Lin, 2021). Therefore, the two types of filial piety corresponding to different parenting styles may also have different effects on gratitude.

Gratitude, whether as a state or a disposition, is in itself prosocial. It encourages individuals to engage in PB in return for the help they receive from others (Bartlett and DeSteno, 2006; Tsang, 2006; Grant and Gino, 2010). For example, Oguni and Otake (2020) found that participants in the experiment group (recalling autobiographical memories of gratitude events) reported more PB than participants in the control group (recalling autobiographical memories of morning routines). A 4-year longitudinal investigation also found that growth in gratitude positively predicted growth in PB (Bono et al., 2019). Based on the above theorizing, we infer that filial piety (RFP and AFP) influences the development of gratitude which serves as a predictor of PB. Thus, Hypothesis 4 was proposed.

*Hypothesis*4: Gratitude mediates the relationship between filial piety (RFP and AFP) and PB.

#### Indebtedness

Indebtedness is an emotional state originating from the norm of reciprocity that makes people who receive favor feel obligated to repay others (Greenberg, 1980). Ho (1994) argued that filial piety emphasizes obedience to parents and therefore can elicit a greater sense of indebtedness. Many studies have also found that compared to individualist societies, such as the United States, East Asian countries (such as Japan and South Korea) that emphasize filial piety tend to have higher levels of indebtedness (Hitokoto et al., 2008; Shen et al., 2011; Kang and Raffaelli, 2016). Therefore, we infer that filial piety can predict a person’s feeling of indebtedness.

Researchers have different views on the influence of indebtedness on PB. Tsang (2007) believes that there is no significant relationship between indebtedness and repaying behavior. Some researchers believe that indebtedness may lead to a lower willingness to repay, which inhibits PB of an individual (Watkins et al., 2006). There are also researchers who believe that indebtedness will prompt an individual to develop moral motivations, leading to more PB (Naito et al., 2005; Peng et al., 2018). Based on findings of recent experimental studies (Naito and Sakata, 2010; Peng et al., 2018), we believe that individuals in a negative mood of indebtedness tend to help others in order to alleviate their own negative feelings (Baumann et al., 1981). Greenberg (1980) also believed that when an individual cannot directly repay the helper, s/he tends to help the people who are similar to the benefactor or people who appear in situations similar to that one receive the favor. This kind of compensation behavior can reduce an individual’s feeling of indebtedness. Based on the above discussion, we put forward the following hypothesis.

*Hypothesis*5: Indebtedness mediates the relationship between filial piety (RFP and AFP) and PB.

### The Moderating Role of Nation

Research on the function of filial piety has not been limited to Confucianism-influenced societies. It has been applied to a broad range of cultural contexts by many scholars (Bedford and Yeh, 2019; Różycka-Tran et al., 2020; Zhou et al., 2020; Bedford and Yeh, 2021). In this study, we are aiming to explore the function of two dimensions of filial piety on prosocial engagement in different nations (China and Indonesia). Culture influences every individual in a unique way (Schwartz, 2013). Therefore, cultural differences can be indicated by the personality, values, and beliefs of individuals from different nations. Here, we used nation rather than culture as a variable for easier understanding. But actually, we are addressing cultural differences rather than national differences. We first made a brief comparative review of the societal and cultural backgrounds in China and Indonesia.

As the core of Confucian collectivist value, filial piety has always been one of the most important moral principles in Chinese society (Bedford and Yeh, 2019). The foundation of filial piety is ancestor worship in ancient China (Hsu, 1975; Bedford and Yeh, 2019). However, with rapid social and economic development, traditional filial piety beliefs in contemporary China are changing (Zhang et al., 2021). Chinese people are becoming more individualistic and self-expressive in the process of urbanization and modernization. Accordingly, filial piety beliefs in China are becoming more reciprocal and less authoritarian. Young people still respect their parents but refuse to completely obey them (Feng, 2013). In Indonesia, however, the traditional cultural values featured by hierarchy and patriarchy (these cultural values are closely associated with AFP) are still prevailing. In this context, obedience and compliance of children to their parents are still highly valued (Riany et al., 2017). Indonesia has the largest Muslim population in the world, with 86.1% of Indonesians considering Islam as their religious belief (Riany et al., 2017). The Islam teachings highly emphasize children’s obedience to and respect for their parents, believing that “obedience to their parents is obedience to God” (Oweis et al., 2012). In addition, Yeh et al. (2013) believed that RFP, which is closely connected with modern democratic values, tends to be more strongly endorsed by women and individuals with higher levels of education and higher levels of socioeconomic status. On the contrary, AFP, which is closely connected with patriarchal values, tends to be more strongly endorsed by males and individuals with lower levels of education and lower levels of socioeconomic status. According to social indicators of economic development, urban population, and education provided by the World Bank (2020), China is relatively more modernized and industrialized than Indonesia. Based on the discussion of cultural values in China and Indonesia, we believe that the Indonesians place more emphasis on parental authority (i.e., AFP) than the Chinese, while the Chinese tend to prefer RFP over AFP.

According to the bioecological model, filial piety, as perceived guiding principles of intergenerational relationships in family and society, can exert direct and indirect impact on people’s psychology and behavior (Bronfenbrenner and Morris, 2006). We believe that the endorsement of different filial piety beliefs in China and Indonesia, as well as religious and cultural values regarding intergenerational relationships, can account for differences in ethical behaviors between the two countries. Bedford and Yeh (2019) posit that the function of RFP tends to show consistency across cultures because RFP is based on feelings deeply rooted in human nature. The function of AFP, however, tends to vary by culture because AFP reflects principles of intergenerational relationships that change with social development. Therefore, we infer that the effects of filial piety (especially AFP) on PB and related mechanisms are different in China and Indonesia.

*Hypothesis*6: The mediating effects of empathy, moral identity, gratitude, and indebtedness in the association of filial piety (RFP and AFP) and PB can be moderated by nation.

## Materials and Methods

### Participants and Procedure

The Chinese and Indonesian participants in this study were both from Guangxi Normal University in Southern China. They were local or international students studying at this university. Indonesian students have studied in China for less than 2years and have not been deeply influenced by Chinese culture. Because we knew in advance that all the participants had no problems in English reading comprehension, English versions of research questionnaires were administered to all of them. The answer time is about 30min, and each person was paid 20 yuan after the investigation. After deleting the invalid cases with unqualified answers or too short response time, the total number of participants was 693 (the initial sample size was 723, with 95.85% were valid cases). Among them, there were 332 Chinese students, with an average age of 20.43years (SD=2.49, ranged from 18 to 30years; 58.4% female); 361 Indonesian students, with an average age of 21.36years (SD=2.81, ranged from 16 to 30years; 45.7% female). This study was conducted in accordance with the 1964 Helsinki declaration and its later amendments or comparable ethical standards and was approved by the academic committee at Guangxi Normal University.

### Measures

#### Dual Filial Piety

Filial piety was measured the Dual Filial Piety Scale (DFPS) compiled by Yeh and Bedford (2003). DFPS contains 16 items, each adopting a six-point Likert scale ranging from 1 (strongly disagree) to 6 (strongly agree). Eight items of DFPS measures RFP (e.g., Be frequently concerned about my parents’ health conditions), and the other eight items measure AFP (e.g., Give up my aspirations to meet my parents’ expectations). The total scores were taken, respectively, with higher scores indicating higher levels of RFP or AFP. In this study, Cronbach’s alpha of DFPS was 0.85 (among Chinese, it was 0.70, and among Indonesians, it was 0.92), while Cronbach’s alpha of the RFP subscale was 0.77 (among Chinese, it was 0.88, and among Indonesians, it was 0.90), and Cronbach’s alpha of the AFP subscale was 0.68 (among Chinese, it was 0.52, and, among Indonesians, it was 0.82).

#### Empathy

The Interpersonal Reactivity Index (IRI; Davis, 1983) was used to assess trait empathy. Based on previous literature (Guo et al., 2019), we selected 22 items (IRI contains 28 items) to measure four dimensions of empathy: perspective-taking (PT), fantasy (FS), empathic concern (EC), and personal distress (PD). Each item (e.g., Imagine how people feel before I criticize them) was rated using a five-point scale from 0 (does not describe me well) to 4 (describes me well). The total score of all items was taken, with a higher score indicating a higher level of empathy. Cronbach’s alpha of IRI in this study was 0.82 (among Chinese, it was 0.80, and among Indonesians, it was 0.84).

#### Moral Identity

Moral identity was evaluated by Moral Identity Measure (MIM) compiled by Aquino and Reed (2002). This measure firstly requires the participants to read a list of nine characteristics (compassionate, fair, caring, friendly, helpful, generous, hardworking, kind, and honest) of a fictional person. Then, they were asked to imagine how people with these characteristics would think, feel, and act. Then, they were further asked to respond to 10 items (e.g., Being a person who has these characteristics makes me feel good) according to their true experiences. Each item is scored using five-point Likert scale (1=strongly disagree, 5=strongly agree). The total score of all items was taken. In this study, Cronbach’s alpha of MIM was 0.84 (among Chinese, it was 0.84, and among Indonesians, it was 0.78).

#### Gratitude

This study used the six-item form of the gratitude questionnaire (GQ-6) compiled by McCullough et al. (2002). A sample item is “I have so much in life to be thankful for.” Participants were asked to answer each item on a scale ranging from 1 (strongly disagree) to 7 (strongly agree). We used the total score of all items for further analysis. Cronbach’s alpha of the gratitude scale in this study was 0.80 (among Chinese, it was 0.83, and among Indonesians, it was 0.72).

#### Indebtedness

We used the Revised Indebtedness scale (IS-R; Bernabé-Valero et al., 2019) to assess the participants’ sense of indebtedness. It was originally developed by Greenberg (1980), containing 22 items ranging from 1 (strongly disagree) to 6 (strongly agree). A sample item is “To owe someone a favor makes me uncomfortable.” The total score of all items was taken. Cronbach’s alpha of IS-R in this study is 0.72 (among Chinese, it was 0.78, and among Indonesians, it was 0.56).

#### Prosocial Behavior

PB is measured by the Self-Reported Altruism Scale Distinguished by the Recipient (SRAS-DR) developed by Oda et al. (2013). SRAS-DR contains a total of 21 items divided into three subscales, measuring PB toward family members, friends, and strangers. Example items are “Kept in tune with one of my family members when they were in a bad mood,” “Phoned or sent an e-mail to a friend who was depressed,” and “Offered help when a stranger was looking for something.” Participants were asked to report how often they participate in PB in their daily lives on a scale ranging from 1 (never) to 5 (very often). This study took the total score of all items. Cronbach’s alpha of SRAS-DR is 0.93 (among Chinese, it was 0.93, and among Indonesians, it was 0.93).

### Data Analysis

In this study, SPSS (version 23) was used for data analysis. We examined the differences in scores between Chinese and Indonesian students on filial piety, empathy, moral identity, indebtedness, gratitude, and PB. The PROCESS macro for SPSS was used for conditional mediation model analysis (Hayes, 2017). Eight models were established based on different independent variables (RFP and AFP) and different mediating variables (empathy, moral identity, gratitude, and indebtedness). We use model 59 in PROCESS to estimate the effects in the moderated mediation models.

## Results

### Preliminary Analyses

Means and SDs of research variables and their correlations in the Chinese and Indonesian samples are presented in Table 1. Among Chinese participants, RFP and AFP were not related, and RFP was positively correlated with PB and other mediating variables, but AFP was only positively correlated with empathy, negatively correlated with gratitude and indebtedness, and was not significantly correlated with PB and moral identity. Among Indonesian participants, RFP and AFP were positively correlated with a coefficient larger than 0.71. These two types of filial piety were positively correlated with all other research variables. In addition, *t* tests showed that the scores of all variables among Chinese and Indonesian participants were significantly different. Except that Chinese participants scored lower on AFP than Indonesian participants, Chinese participants had higher scores than Indonesian participants on other variables.

**Table 1:** Means, SDs, and correlations in different nations (*n*=693).

	1	2	3	4	5	6	7
1.RFP	1	0.71^***^	0.39^***^	0.57^***^	0.50^***^	0.35^***^	0.35^***^
2.AFP	−0.02	1	0.41^***^	0.41^***^	0.44^***^	0.16^**^	0.35^***^
3.Empathy	0.27^***^	0.13^*^	1	0.29^***^	0.32^***^	0.08	0.34^***^
4.Moral Identity	0.55^***^	−0.02	0.38^***^	1	0.54^***^	0.44^***^	0.37^***^
5.Gratitude	0.62^***^	−0.20^***^	0.26^***^	0.58^***^	1	0.39^***^	0.52^***^
6.Indebtedness	0.54^***^	−0.12^*^	0.14^*^	0.48^***^	0.65^***^	1	0.30^***^
7.PB	0.66^**^	0.02	0.32^**^	0.61^**^	0.64^**^	0.53^**^	1
China (*n*=332)							
Mean	39.64	28.69	75.51	38.98	33.36	82.29	84.60
SD	6.25	4.72	10.88	5.91	6.23	10.38	12.82
Indonesia (*n*=361)							
Mean	36.90	32.76	69.52	33.28	30.59	77.24	78.50
SD	7.30	6.28	10.72	5.55	4.81	6.87	12.24
*t*	5.31^***^	−9.68^***^	7.29^***^	13.09^***^	6.50^***^	7.47^***^	6.41^***^

Correlations for Indonesia are above the diagonal, while correlations for China are below the diagonal; PB, prosocial behavior; the *t* test for the scores of participants from both nations on each variable is presented in the last row.

* *p*<0.05;

** *p*<0.01;

*** *p*<0.001.

### Testing Mediation and Moderation Effects

**Figure 1:**
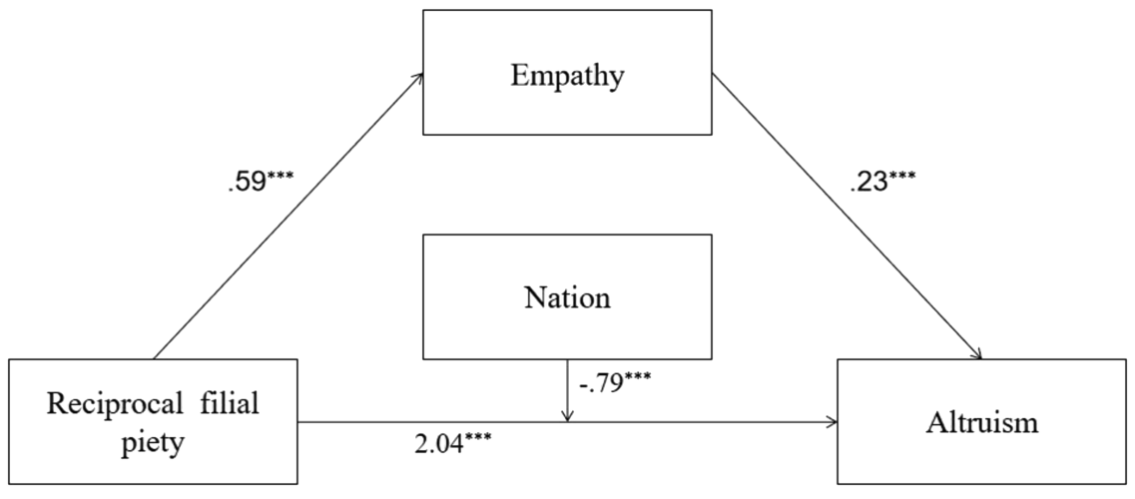
Mediation and moderation model showing standardized coefficients (Model 1); ^***^p<0.001.

Since Models 1 to 4 are not conditional mediation models, we tested them separately. In Model 1 of Table 2, it showed that RFP predicted empathy, and RFP explained 14% of empathy’s total variance [*R*^2^=0.14, *F*(1,691)=108.32, *p*=0.00]. At the same time, RFP, empathy, and nation all predicted PB, and the interaction between RFP and nation was significant. Nation moderated the direct effect of RFP on PB. All predictors explain 35% of the total variance of PB [*R*^2^=0.35, *F*(4,688)=93.93, *p*=0.00]. In addition, the indirect effect of RFP on PB was significant. That is, the mediating effect of empathy was significant. Detailed descriptions of Model 1 are shown in Figure 1.

**Table 2:** Testing the mediation and moderation effects.

Predictors	*β*	SE	*t*	*p*	95% CI
Model 1:						Outcome: empathy
RFP	0.59	0.06	10.41	0.00	(0.48, 0.71)
Outcome: PB					
RFP	2.04	0.20	10.30	0.00	(1.65, 2.43)
Empathy	0.23	0.04	5.79	0.00	(0.15, 0.30)
Nation	27.79	4.64	5.99	0.00	(18.67, 36.90)
RFP * Nation	−0.79	0.12	−6.67	0.00	(−1.02, −0.56)
Conditional direct effects					
China	1.25	0.09	13.44	0.00	(1.07, 1.44)
Indonesia	0.46	0.08	5.91	0.00	(0.31, 0.62)
Indirect effect of X on Y					
Empathy	0.13	0.02			(0.09, 0.18)
Model 2:						Outcome: moral identity					
RFP	0.53	0.03	18.57	0.00	(0.47, 0.59)
Outcome: PB					
RFP	1.71	0.20	8.59	0.00	(1.32, 2.11)
Moral identity	0.67	0.08	8.22	0.00	(0.51, 0.83)
Nation	26.50	4.52	5.86	0.00	(17.62, 35.39)
RFP * Nation	−0.71	0.12	−6.09	0.00	(−0.93, −0.48)
Conditional direct effects					
China	1.01	0.10	10.19	0.00	(0.81, 1.20)
Indonesia	0.30	0.08	3.71	0.00	(0.14, 0.46)
Indirect effect of X on Y					
Moral identity	0.36	0.06			(0.24, 0.47)
Model 3:						Outcome: gratitude
RFP	0.47	0.03	18.14	0.00	(0.42, 0.52)
Outcome: PB					
RFP	1.27	0.20	6.35	0.00	(0.87, 1.66)
Gratitude	0.95	0.08	11.59	0.00	(0.79, 1.11)
Nation	16.79	4.40	3.81	0.00	(8.15, 25.44)
RFP * Nation	−0.49	0.11	−4.35	0.00	(−0.71, −0.27)
Conditional direct effects					
China	0.77	0.10	7.78	0.00	(0.58, 0.97)
Indonesia	0.28	0.08	3.73	0.00	(0.13, 0.43)
Indirect effect of X on Y					
Gratitude	0.44	0.05			(0.34, 0.55)
Model 4:						Outcome: empathy
AFP	0.32	0.07	4.58	0.00	(0.19, 0.46)
Outcome: PB					
AFP	−0.58	0.29	−2.00	0.04	(−1.15, −0.01)
Empathy	0.95	0.08	11.59	0.00	(0.79, 1.11)
Nation	−20.96	5.20	−4.03	0.00	(−31.18, −10.74)
AFP * Nation	0.52	0.17	3.09	0.00	(0.19, 0.85)
Conditional direct effects					
China	−0.06	0.14	−0.43	0.67	(−0.33, 0.21)
Indonesia	0.46	0.10	4.51	0.00	(0.26, 0.66)
Indirect effect of X on Y					
Empathy	0.11	0.03			(0.06, 0.16)

AFP, authoritarian filial piety; Nation=1 (Chinese) or 2 (Indonesian); PB, prosocial behavior; RFP, reciprocal filial piety.

**Figure 2:**
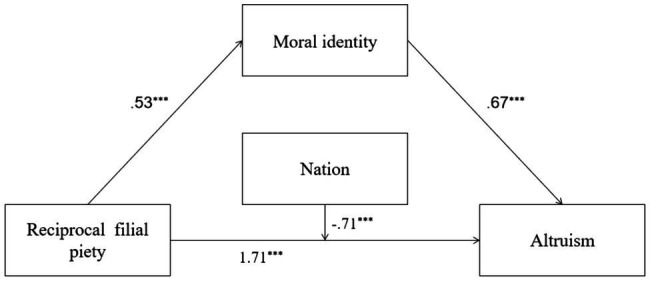
Mediation and moderation model showing standardized coefficients (Model 2); ^***^p<0.001.

In Model 2, a higher level of RFP was associated a higher level of moral identity, and the total variance of moral identity explained by RFP was 33% [*R*^2^=0.33, *F*(1,691)=344.91, *p*=0.00]. RFP, moral identity, and nation all predicted PB, and the interaction between RFP and nation was also significant. Nation moderated the direct effect of RFP on PB, and the total variance of PB explained by all predictors was 38% [*R*^2^=0.38, *F*(4,688)=106.47, *p*=0.00]. Moral identity’s mediating role was also significant. Detailed descriptions of Model 2 are shown in Figure 2.

**Figure 3:**
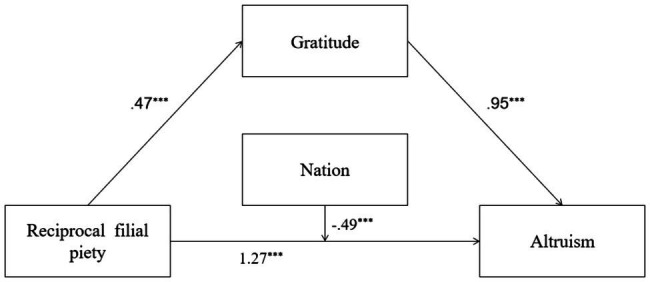
Mediation and moderation model showing standardized coefficients (Model 3); ^***^p<0.001.

In Model 3, RFP positively and significantly predicted gratitude, explaining 32% of the total variance of gratitude [*R*^2^=0.32, *F*(1,691)=328.88, *p*=0.00]. RFP, gratitude, and nation significantly predicted PB, and the interaction between RFP and nation was also significant. The predictors explained 43% of PB’s total variance [*R*^2^=0.43, *F*(4,688)=131.12, *p*=0.00]. Gratitude played a mediating role in the relation between RFP and PB. Detailed descriptions of Model 3 are shown in Figure 3.

**Figure 4:**
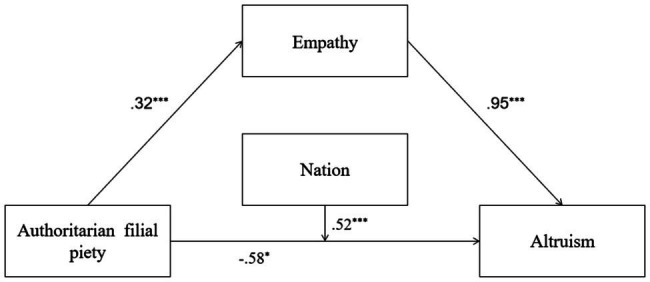
Mediation and moderation model showing standardized coefficients (Model 4); ^*^p<0.05; ^***^p<0.001.

In Model 4, AFP predicted empathy positively. AFP only explained 3% of the total variance of empathy [*R*^2^=0.03, *F*(1,691)=21.00, *p*=0.00]. AFP and nation negatively predicted PB, while empathy positively predicted PB. The interaction between AFP and nation was significant. The predictors explained 18% of the total variance of PB [*R*^2^=0.18, *F*(4,688)=38.47, *p*=0.00]. The AFP of Indonesian students directly predicted PB, but the AFP of Chinese students did not. Empathy mediated the connection between AFP and PB. Detailed descriptions of Model 4 are shown in Figure 4.

### Testing for the Moderated Mediation Effects

**Figure 5:**
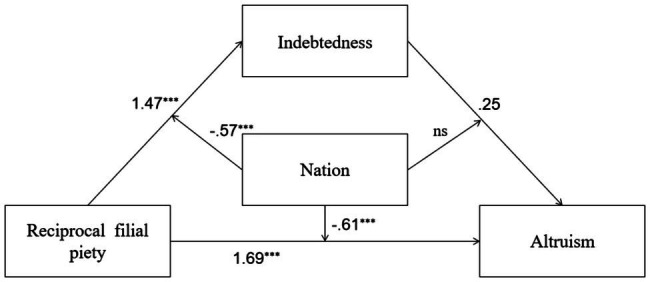
Moderated mediation model showing standardized coefficients (Model 5); ^***^p<0.001.

In Table 3, we conducted a conditional mediation model analysis for Models 5 to 8 with RFP/AFP as the independent variable and indebtedness, moral identity, or gratitude as the mediating variable. In Model 5, a higher level of RFP was associated with a higher level of indebtedness; nation positively predicted indebtedness, and the interaction between RFP and nation was also significant. All predictors explained 30% of the total variance of indebtedness [*R*^2^=0.30, *F*(3,689)=96.39, *p*=0.00]. At the same time, both RFP and nation positively predicted PB, and indebtedness did not predict PB. And the interaction between RFP and nation was significant, while the interaction between indebtedness and nation was not significant. The total variance of PB explained by all predictors was 36% [*R*^2^=0.36, *F*(5,687)=77.08, *p*=0.00]. In this model, the indirect effects in the two countries were both significant, which need to be further tested. Detailed descriptions of Model 5 were shown in Figure 5.

**Table 3:** Testing for moderated mediation.

Predictors	*β*	SE	*t*	*p*	95% CI
Model 5						Outcome: indebtedness
RFP	1.47	0.15	10.12	0.00	(1.18, 1.76)
Nation	18.51	3.40	5.44	0.00	(11.82, 25.19)
RFP*Nation	−0.57	0.09	−6.57	0.00	(−0.74, −0.40)
Outcome: PB					
RFP	1.69	0.23	7.34	0.00	(1.24, 2.15)
Indebtedness	0.25	0.16	1.58	0.11	(−0.06, 0.55)
Nation	16.36	7.80	2.10	0.04	(1.04, 31.68)
Indebtedness * Nation	0.06	0.11	0.55	0.58	(−0.15, 0.27)
RFP * Nation	−0.61	0.13	−4.54	0.00	(−0.87, −0.35)
Conditional indirect effects					
China	0.27	0.06			(0.17, 0.39)
Indonesia	0.12	0.04			(0.04, 0.22)
Model 6:						Outcome: moral identity
AFP	−0.42	0.14	−3.07	0.00	(−0.68, −0.15)
Nation	−18.30	2.41	−7.58	0.00	(−23.04, −13.56)
AFP * Nation	0.39	0.08	4.92	0.00	(0.23, 0.54)
Outcome: PB					
AFP	−0.31	0.27	−1.16	0.25	(−0.83, 0.21)
Moral identity	2.06	0.23	9.06	0.00	(1.61, 2.50)
Nation	12.54	6.57	1.91	0.06	(−0.35, 25.43)
Moral identity * Nation	−0.73	0.15	−4.89	0.00	(−1.02, −0.43)
AFP * Nation	0.39	0.16	2.47	0.01	(0.08, 0.70)
Conditional indirect effects					
China	−0.04	0.10			(−0.24, 0.15)
Indonesia	0.22	0.06			(0.11, 0.35)
Model 7:						Outcome: gratitude
AFP	−0.86	0.13	−6.63	0.00	(−1.12, −0.61)
Nation	−21.39	2.31	−9.27	0.00	(−25.93, −16.86)
AFP * Nation	0.60	0.08	7.97	0.00	(0.45, 0.75)
Outcome: PB					
AFP	0.51	0.26	1.99	0.04	(0.01, 1.02)
Gratitude	1.60	0.22	7.31	0.00	(1.17, 2.03)
Nation	6.34	6.23	1.02	0.31	(−5.90, 18.58)
Gratitude * Nation	−0.22	0.15	−1.45	0.15	(−0.52, 0.08)
AFP * Nation	−0.11	0.15	−0.71	0.48	(−0.41, 0.19)
Conditional indirect effects					
China	−0.36	0.12			(−0.59, −0.14)
Indonesia	0.39	0.07			(0.26, 0.54)
Model 8:						Outcome: indebtedness
AFP	−0.68	0.21	−3.15	0.00	(−1.10, −0.25)
Nation	−17.88	3.81	−4.69	0.00	(−25.36, −10.40)
AFP * Nation	0.42	0.12	3.40	0.00	(0.18, 0.67)
Outcome: PB					
AFP	−0.19	0.27	−0.69	0.49	(−0.73, 0.35)
Indebtedness	0.89	0.14	6.13	0.00	(0.60, 1.17)
Nation	0.09	9.37	0.01	0.99	(−18.30, 18.49)
Indebtedness * Nation	−0.22	0.10	−2.11	0.04	(−0.42, −0.01)
AFP * Nation	0.40	0.16	2.52	0.01	(0.09, 0.71)
Conditional indirect effects					
China	−0.17	0.08			(−0.33, −0.01)
Indonesia	0.08	0.04			(0.02, 0.16)

AFP, authoritarian filial piety; Nation=1 (Chinese) or 2 (Indonesian); PB, prosocial behavior; RFP, reciprocal filial piety.

**Figure 6:**
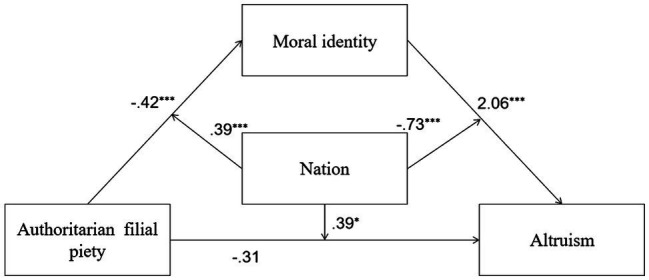
Moderated mediation model showing standardized coefficients (Model 6); ^*^p<0.05; ^***^p<0.001.

In Model 6, both AFP and nation significantly predicted moral identity, and the interaction between AFP and nation was also significant. The total variance of moral identity explained by these predictors was 26% [*R*^2^=0.26, *F*(3,689)=82.28, *p*=0.00]. At the same time, AFP and nation did not predict PB, but moral identity predicted PB, and the interaction between moral identity and nation was significant, and the interaction between AFP and nation was significant too. The total variance of PB explained by all predictors was 32% [*R*^2^=0.32, *F*(5,687)=65.29, *p*=0.00]. The indirect effect of AFP on PB through moral identity was significant in Indonesian students, but not significant in Chinese students, indicating that the moderated mediation model was supported. Detailed descriptions of Model 6 are shown in Figure 6.

**Figure 7:**
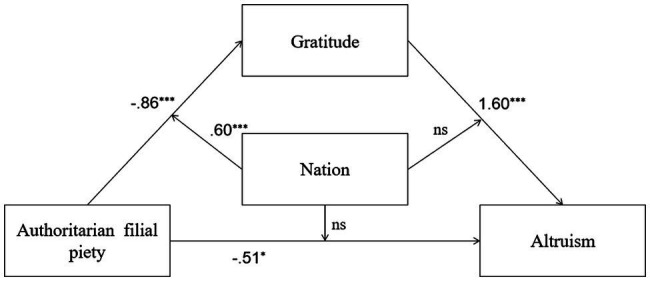
Moderated mediation model showing standardized coefficients (Model 7); ^*^p<0.05; ^***^p<0.001.

In Model 7, both AFP and nation significantly and negatively predicted gratitude, and the interaction between AFP and nation was also significant. All predictors explained 15% of the total variance of gratitude [*R*^2^=0.15, *F*(3,689)=41.82, *p*=0.00]. In addition, both AFP and gratitude positively predicted PB, while nation did not predict PB. The interaction between AFP and nation was not significant. The interaction between gratitude and the nation was not significant too. These predictors explained 40% of the total variance of PB [*R*^2^=0.40, *F*(5,687)=90.34, *p*=0.00]. In this model, the conditional indirect effects were significant. Therefore, further analysis of this model was conducted. Detailed descriptions of Model 7 are shown in Figure 7.

**Figure 8:**
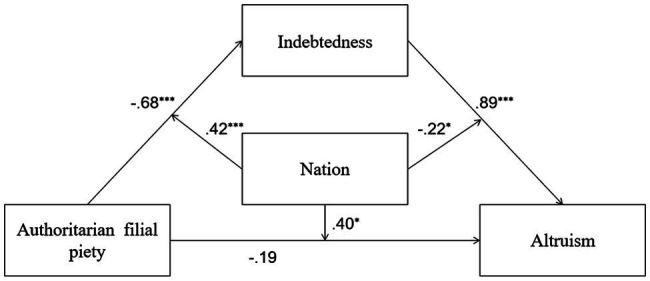
Moderated mediation model showing standardized coefficients (Model 8); ^*^p<0.05; ^***^p<0.001.

In Model 8, both AFP and nation significantly predicted indebtedness, and the interaction between AFP and nation was also significant. All predictors explained 9% of the total variance of indebtedness [*R*^2^=0.09, *F*(3,689)=23.41, *p*=0.00]. At the same time, AFP and nation did not predict PB, but indebtedness predicted PB, and the interaction between indebtedness and nation was significant, and the interaction between AFP and nation was significant. All predictors explained 28% of the total variance of PB [*R*^2^=0.28, *F*(5,687)=53.73, *p*=0.00]. The conditional indirect effects of this model are also significant. Therefore, further analysis of this model was conducted. Detailed descriptions of Model 8 are shown in Figure 8.

Since the indirect effects of some models were significant in both China and Indonesia, it is impossible to directly determine whether the moderated mediation model holds. Therefore, according to Hayes (2015), we used the index for moderated mediation to further determine whether the conditional mediation model is supported. The indices of moderated mediation are listed in Table 4. All CI did not contain zero, indicating that these conditional mediation models were all supported.

**Table 4:** Indices of moderated mediation.

Models	Index	SE	95% CI
Model 5	−0.15	0.07	(−0.30, −0.01)
Model 6	0.26	0.11	(0.04, 0.49)
Model 7	0.75	0.14	(0.49, 1.02)
Model 8	0.25	0.09	(0.07, 0.43)

## Discussion

Researchers believe that filial piety is a universal contextualized personality construct that can be applied to parent-child relationships in a global context (Bedford and Yeh, 2019; Beckert et al., 2020). Based on DFPM, we tested the similarities and differences in the psychological functions of RFP/AFP in Chinese and Indonesian students. First, we observed different RFP and AFP scores in the two samples. As expected, individuals in China, a more industrialized and modernized society, had higher RFP levels, while participants who grew up in Indonesia, a society still emphasizes hierarchies, had higher AFP levels (Yeh et al., 2013; Riany et al., 2017). Another interesting phenomenon is the difference in correlation between the RFP and AFP in the two countries. In China, the correlation coefficient between RFP and AFP was not significant, but RFP and AFP of Indonesian students showed a high correlation. In China, the distinction between RFP and AFP can be explained by increased individualism and the impact of rapid modernization. The core of filial piety has changed from absolute obedience to parental authority to equality and mutual care in parent-child relationships (Yeh et al., 2013). Indonesian society emphasizes authority and obedience, coupled with the influence of Islam (Oweis et al., 2012; Riany et al., 2017), which may be responsible for why different dimensions of filial piety are connected rather than separated.

In addition, this study investigated the relationships between key research variables among Chinese and Indonesians, as well as the potential moderated mediation effects. Consistent with Hypothesis 1a, RFP positively and significantly predicted PB in all models, indicating that individuals with higher RFP levels are more likely to engage in PB. This finding links RFP with PB and verified that RFP has similar functions in different cultures (Bedford and Yeh, 2019). But unlike Hypothesis 1b, we found contradictory results regarding the function of AFP. The results of model 4 showed that AFP significantly and negatively predicted PB, and AFP in model 7 positively predicted PB, but in models 6 and 8, AFP did not predict PB. In Table 1, we found that the correlation between AFP and PB was only 0.02 among Chinese participants, while the correlation between AFP and PB was positive and significant among Indonesian participants. We believe that this contradictory result is caused by the cultural differences between the two countries, which will be discussed in the next model.

Consistent with Hypothesis 2, the results of model 1 and model 4, respectively, confirmed the influence of RFP and AFP on PB through empathy (i.e., RFP/AFP→empathy→PB). It is easy to understand that high RFP individuals are more likely to empathize with others’ misfortunes and take others’ perspectives (Yeh and Bedford, 2003), and high empathic ability is associated with stronger motivation to help others (Telle and Pfister, 2015; Van der Graaff et al., 2018). It is worth noting that nation did not moderate the indirect effect of RFP on PB through empathy, but moderated the direct effect of RFP on PB. RFP can directly promote PB in two countries, but the effect is greater in China (compared to Indonesia). Generally to say, the function of RFP showed more similarities than differences in the two countries.

But when the predictor was AFP (see model 4), the result was exactly the opposite. Nation also did not moderate the influence of AFP on PB through empathy but moderated the direct effect of AFP on PB. As to the conditioned direct effect, Indonesian AFP can positively and significantly predict PB, while Chinese AFP did not significantly predict PB. This reflects that the function of AFP in the two countries is completely opposite.

In consistent with Hypothesis 3, both RFP and AFP can affect PB through moral identity. This study found that filial piety, as an important component of family factors, can predict a person’s moral identity. And moral identity can stimulate moral motivation, thereby promoting PB (Aquino and Reed, 2002; Hardy, 2006). But this indirect link showed national differences. When the predictor was RFP, its positive influence on PB *via* moral identity was not moderated by nation. However, the direct effect of RFP on PB was stronger among Chinese (compared to Indonesian) participants. The situation was reversed when AFP was the predictor. Among Chinese participants, AFP did not directly and indirectly (*via* moral identity) predict PB (model 6). Among Indonesian participants, AFP positively predict PB and did so indirectly *via* moral identity.

In consistent with Hypothesis 4, two types of filial piety (RFP and AFP) can influence PB through gratitude. Gratitude is firstly developed in family and can predict a person’s PB in other interpersonal settings (Scabini et al., 2006; Bono et al., 2019). This study found that gratitude played a mediating role in the association of RFP and PB and this mediating effect did not differ by nation. However, the mediating effect of gratitude in the association of RFP and PB was positive among Indonesian participants, but negative among Chinese participants.

In consistent with Hypothesis 5, indebtedness mediated the relationship between filial piety (RFP and AFP) and PB. Filial piety, as an important Confucian virtue, emphasizes the importance of obeying and repaying the parents (Kang and Larson, 2014; Bedford and Yeh, 2019). This suggests that filial piety can enhance the sense of indebtedness (Kang and Larson, 2014). This study successfully established a connection between filial piety and PB through indebtedness. Indebtedness could promote PB directly in two nations, with a stronger positive effect observed among Chinese (relative to Indonesian) participants. Additionally, the mediating effect of indebtedness was also stronger among Chinese (relative to Indonesian) participants. The results turned out to be complicated again when AFP was the predictor. Indonesian AFP positively predicted PB through indebtedness, but Chinese AFP negatively predicted PB through indebtedness.

Partly consistent with Hypothesis 6, national culture moderated the influence of AFP on PB through various mediating variables (moral identity, gratitude, and indebtedness) and also moderated the influence of RFP on PB through indebtedness. Inconsistent with Hypothesis 6, national culture did not moderate the influence of RFP on PB through empathy, moral identity, or gratitude nor did it moderate the influence of AFP on PB through empathy. But it moderated the direct effects of RFP/AFP on PB in these three models. This also reflects the similarities across cultures in the functions of RFP which is based on the emotional connection (Bedford and Yeh, 2019). In this study, we argue that AFP has a greater impact on PB than RFP in a society that places relatively more emphasis on hierarchy and obedience, while in a more modernized and industrialized society where individual well-being and self-expression are more emphasized, RFP has a stronger effect on PB.

## Limitations and Future Directions

There are some limitations expected to be solved by future researches. First, Tsao and Yeh (2019) believe that the cross-cultural study of filial piety should focus on identifying cultural similarities and differences in the psychological functions of RFP and AFP. Although this study has explored the similarities and differences in the roles of RFP/AFP in China and Indonesia, it has not explored the reasons and mechanisms in depth. Mutual relationships and possible interactions between four mediators, which may be important in understanding how filial piety influences PB, were not assumed in this study. Further research can make further exploration on how cultural factors condition the mechanisms of RFP and AFP. Second, we only tested the similarities and differences in the functions of RFP and AFP and the influential mechanism in two cultures (China and Indonesia). It is not known whether the results of this study can be extended to more cultures. This should be addressed in a broader range of cultural contexts. Third, this study cannot determine causality due to using a cross-sectional design. Future research can consider the use of experimental or longitudinal methods to confirm causal links. Additionally, participants in this study are all college students that cannot represent the general population of a country. Future research can overcome this limitation by using samples of different occupational and age groups.

Despite these limitations, this study is the first to examine relevant mechanisms in the relationships between different dimensions of dual filial piety and PB. We found that RFP and AFP affect PB in different ways and are moderated by national culture. This made an advance in understanding the functions of filial piety in different cultures. RFP can consistently promote PB in China and Indonesia *via* the cultivation of several character strengths, while AFP can do so only in Indonesia. In a modernized society like China, parents should adopt a more humanitarian manner to cultivate their children’s RFP to promote their prosocial development. In a more hierarchical society like Indonesia, it seems both RFP and AFP are encouraged to be cultivated to promote prosocial development. However, we still believe that parents should consider cultivating children’s RFP more than AFP to promote the development of positive psychological outcomes.

## Data Availability Statement

The raw data supporting the conclusions of this article will be made available by the authors, without undue reservation.

## Ethics Statement

The studies involving human participants were reviewed and approved by the Academic Committee at Guangxi Normal University. The patients/participants provided their written informed consent to participate in this study.

## Author Contributions

WZ analyzed the data, wrote the original manuscript, and revised the manuscript. QG designed the work, provided data analysis ideas, and revised the manuscript. TH and JL helped to revise the manuscript. CX provided data analysis software. All authors contributed to the article and approved the submitted version.

## Conflict of Interest

The authors declare that the research was conducted in the absence of any commercial or financial relationships that could be construed as a potential conflict of interest.

## Publisher’s Note

All claims expressed in this article are solely those of the authors and do not necessarily represent those of their affiliated organizations, or those of the publisher, the editors and the reviewers. Any product that may be evaluated in this article, or claim that may be made by its manufacturer, is not guaranteed or endorsed by the publisher.
